# Decision-Making in Multiple Sclerosis Patients: A Systematic Review

**DOI:** 10.1155/2018/7835952

**Published:** 2018-03-12

**Authors:** Mireille Neuhaus, Pasquale Calabrese, Jean-Marie Annoni

**Affiliations:** ^1^Unit of Neurology, University and Hospital of Fribourg, Fribourg, Switzerland; ^2^Neuropsychology and Behavioral Neurology Unit, Faculty of Psychology and Interdisciplinary Platform Psychiatry and Psychology, Division of Molecular and Cognitive Neuroscience, University of Basel, Basel, Switzerland; ^3^Department of Neurology, University Hospital of Basel, Basel, Switzerland; ^4^Department of Clinical Neurosciences, University and Hospital of Geneva, Geneva, Switzerland

## Abstract

**Background:**

Multiple sclerosis (MS) is frequently associated with cognitive and behavioural deficits. A growing number of studies suggest an impact of MS on decision-making abilities. The aim of this systematic review was to assess if (1) performance of MS patients in decision-making tasks was consistently different from controls and (2) whether this modification was associated with cognitive dysfunction and emotional alterations.

**Methods:**

The search was conducted on Pubmed/Medline database. 12 studies evaluating the difference between MS patients and healthy controls using validated decision-making tasks were included. Outcomes considered were quantitative (net scores) and qualitative measurements (deliberation time and learning from feedback).

**Results:**

Quantitative and qualitative decision-making impairment in MS was present in 64.7% of measurements. Patients were equally impaired in tasks for decision-making under risk and ambiguity. A correlation to other cognitive functions was present in 50% of cases, with the highest associations in the domains of processing speed and attentional capacity.

**Conclusions:**

In MS patients, qualitative and quantitative modifications may be present in any kind of decision-making task and can appear independently of other cognitive measures. Since decision-making abilities have a significant impact on everyday life, this cognitive aspect has an influential importance in various MS-related treatment settings.

## 1. Introduction

Decisions are a challenge we all face daily. Deficits in the ability to make decisions can have far reaching consequences on all aspects of our lives, such as finances and social interactions.

Decision-making is a complex behavioural process requiring multiple steps (see Ernst and Paulus [1] for a comprehensive review).

First, by realizing that a decision must be taken, individuals initiate an internal process that starts with the definition of the nature of the decision that has to be made. Second, mental search and imaginative abilities are crucial. If the probabilities for possible outcome scenarios are given but the outcome is not, then subjects have to decide under risk. In case that neither outcome, nor the probabilities for specific outcomes are known, decisions are taken under ambiguity. In a next step, the collected information is weighted against emotions. Hence, decision-making is driven by cognitive as well as emotional components [2]. Finally, once a decision has led to unfavourable results, it has to be evaluated concerning its long-term consequences. Thus, besides the mere decisional components (e.g., selecting and weighing) attentional, mnestic, executive, and prospective thinking and learning abilities are required, for example, for the capacity to modify or to adapt to wrong choices. Decisions under ambiguity show little correlation with executive functions [3] and an asymmetrical dependency on working memory. This means that isolated deficits in decision-making with preserved working memory performance are possible while impaired working memory consistently leads to a deficit in decision-making [4]. In contrast to this, impairments in tasks with decisions under risk (such as dice games) have been correlated to specific executive functions such as categorization, monitoring, and using of feedback for current decisions [5, 6].

Emotional components also exert a significant influence in decision-making performance. Damasio's somatic marker hypothesis [7], which introduces an emotional component to the decision-making process states that, faced with a decision, a first selection of the most advantageous choices is made by balancing positive and negative “somatic markers,” measured by skin conductance reaction [8] in gambling tasks. Emotional markers apply to every decision, such as choosing clothes, profession, or accepting a medical treatment. However, most paradigms come from neuroeconomy.

Given this cognitive and emotional complexity, the process of decision-making has been associated with a large corticosubcortical network encompassing different structures of the ventral prefrontal cortex [8], specifically the ventromedial [2] areas, and the orbitofrontal cortex [9, 10], but also the amygdala and the striatal system [11].

Multiple sclerosis (MS) is a demyelinating disease of the central nervous system, which affects both white and grey matter [12]. Cognitive domains such as attention, information-processing speed and efficiency, executive functioning, and long-term memory are known to be affected in 43–70% of all patients, in early as well as in late stages of the disease (for review see [13, 14]). Modifications of emotional experience and verbalization have been shown in MS patients [15]. Most recently, a growing field of research has accumulated, reporting deficits or alterations in decision-making in MS patients [16–26].

As MS patients have to make decisions about their treatment and lifestyle, it is of eminent importance to know, whether and if so, how their decision-making abilities may be subjected to changes due to their disease.

The aim of this paper is to review the existing literature on decision-making in MS patients and to answer the following questions.

(1) Is modified decision-making in MS patients a consistent finding in pertinent studies? If so, what is the nature of this modification? Is there a difference between decision-making performance under risk and under ambiguity? (2) Is modified decision-making associated with cognitive dysfunction in particular domains or altered emotional reactions?

## 2. Methods

### 2.1. Article Selection

Between 1 and 13 May 2015, we searched the Medline/Pubmed database for articles published from 2004 to 2015, using the keywords: “Decision-making”, “Multiple Sclerosis”, “Cognition”, “Gambling” and “Dice”. We selected for a thorough analysis all original studies that fitted our inclusion criteria. Additional papers that fitted the inclusion criteria and were published after the search period were also included.

### 2.2. Inclusion Criteria

To qualify for inclusion in our analysis, the studies had to fulfil the following criteria: (i) they had to comprise ten or more participants in the MS patients group. (ii) The patient's decision-making performances had to be compared against a healthy control group without neurological or psychiatric disorders. (iii) The assessment of decision-making abilities was performed with explicitly described and clinically validated tasks.

### 2.3. Data Collection and Analysis

We recorded the results of the decision-making tests and, whenever assessed in the study, associated values in general cognition or executive performance and mood and behavioural tests. When several decision-making tests were applied in one study, each measurement was considered separately. Differences in DM performance between MS patients and controls were considered significant if the results of the reported net scores were lower by adopting an error probability of *p* < 0.05 in the patients group compared to healthy controls. Where no net scores were stated differences in qualitative measurements such as deliberation time and risk adjustments were considered for the description of global performance. The same qualitative measures were considered to unravel possible differences in the learning process between MS patients and controls.

### 2.4. Tasks Used in the Selected Clinical Studies

#### 2.4.1. Decision under Risk Tasks

In the Game of Dice Task (GDT) [5] the subjects have to maximize the winnings by guessing which number a virtual dice will show next. They can predict one number, in which case possible gains but also losses are high. They can reduce the risk by choosing a combination of up to four numbers, which increases the possibility of winning but also decreases the possible gains and losses. Choices of one or two dice are considered risky/disadvantageous (less than 50% winning probabilities); choices of three or four numbers are considered advantageous. In total, the die is thrown 18 times. This task was developed to minimise the ambiguity of the gambling situation by establishing explicit and stable rules. Quality of decision-making is measured using a total net score calculated by extracting the number of disadvantageous choices from the number of advantageous ones.

In the Cambridge Gambling Task (CGT) [10], subjects have to maximize their winnings by guessing if a token is hidden under a red or a blue box. While the total number of boxes remains constant, the ratio of the colours varies from round to round and so do the associated stakes. Correct guesses are rewarded; wrong choices punished with an amount chosen beforehand by the participant. The main outcome is the proportion of trials in which the participant chooses the more likely outcome. Other measured values are deliberation time for each decision, amount bet, an impulsivity index, and the risk adjustment (the adjustment of bet amount to the stakes). In some studies, the total of blocks ending up in bankruptcy was also calculated.

In the Wheel of Fortune task (WOF) [29], two wheels appear on a computer screen, each of them is divided into two sectors. Each sector is associated with a positive or negative value, corresponding to the amount added or subtracted from the total winnings, should the needle, after rotating, stop in this sector. The sectors size indicates the probability of the outcome. The subjects have to choose one wheel. The aim is, again, to maximize the winnings. The quality of decision-making was assessed by the maximization of expected values. Other measurements included the emotional evaluation of outcomes and the effect of anticipating disappointment and regret.

#### 2.4.2. Decisions under Ambiguity Tasks

In the Iowa Gambling Task (IGT) [30], the subjects are presented with four decks of cards (decks A–D). They have to turn the cards, one by one. Cards from the decks A and B are consistently associated with a winning of 100 dollars or more in play money and decks C and D with lower incomes (in the order of 50 dollars). After having turned a certain number of cards, the subjects additionally receive irregularly an apparently unpredictable punishment. In fact high winnings are associated with even higher punishment, resulting in total losses, whereas low winnings are associated with even lower punishment, thus resulting in a positive balance. A total of 100 choices are to be made. These rules are not explained to the subjects, who have to gradually identify the nature of the decks and switch to choosing the advantageous cards. The index of performance is obtained by subtracting the number of disadvantageous choices (cards from blocks A and B) from the advantageous choices (cards from blocks C and D). For the net score, all trials are considered. Some studies also analysed the progression of choices by dividing the task into 5 trials, each consisting of 20 choices and calculating the index of performance for each trial separately.

#### 2.4.3. Cognitive Function Tasks

In the included studies, the following tests were used to assess cognitive domains other than decision-making (Table 1).

#### 2.4.4. Assessment of Mood, Behaviour, and Quality of Life

The following tests were used to assess the subject's mood, behaviour, and quality of life: Dysexecutive Questionnaire (DEX); Iowa Scale of Personality Change (ISPC); Fatigue: Fatigue Assessment Inventory (FAI); Hospital Anxiety and Depression Scale (HAD); Handicap: London Handicap Scale; Self-Perceived Health (SEP-59); Beck Depression Inventory (BDI).

### 2.5. Statistical Analysis

The proportion of studies showing a significant difference in net decision-making scores between MS patients and controls among the total number of reviewed studies is presented in raw data, *z*-score, and percentage. For the comparison of results between two groups (tasks under risk and under ambiguity, EDSS </>3), the *χ*^2^ test was used.

## 3. Results

### 3.1. Sample Characteristics

A total of 12 studies matched the inclusion criteria and were therefore considered for the study. Sample size varied from *n* = 12 to *n* = 165 MS/Clinically Isolated Syndrome (CIS) patients with an mean EDSS (expanded disability status scale [31]) ranging from 1.03 to 7.2.  Table 2 shows clinical and neuropsychological data of the patient groups.

#### 3.1.1. Decision-Making in MS and CIS Patients

In total, 17 tasks for decision-making (DM) were measured and in each study the performance was compared to healthy controls. Performance of MS patients was impaired compared to the healthy controls in 11/17 tasks (64.7%) and preserved in 6/17 tasks (35.3%).

**Figure 1:**
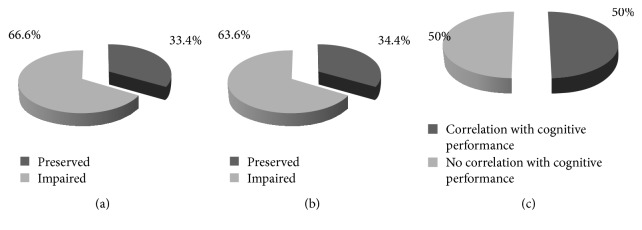
Proportions of preserved and impaired performance in decisions under risk tasks (a) and under ambiguity (b). Proportion of measurements showing a correlation to the performance in other cognitive tasks (c).

*(a) Decision-Making under Risk in MS Patients*. As described above, the tasks GDT, CGT, and WOF are considered to test decision-making under risk. In total, decision-making under risk was impaired in MS patients in 4/6 (66.6%) over all analysed measurements (cf.  Figure 1(a)). 

*(b) Decision-Making under Ambiguity*. All analysed studies reported a total of 11 measurements of decision-making under ambiguity, all of them using the Iowa Gambling Task. Based on the net score, performance of MS patients was impaired in 7/11 (63.6%) of the tests (cf.  Figure 1(b)).

*χ* ^2^ showed no difference between the performance of MS patients in the two task groups (under ambiguity/under risk) (*p* = 0.79). 

*(c) Qualitative Changes in DM*. Qualitative analysis of DM included mostly two groups of measurements: adjustment to feedback and deliberation time. Subjects were considered to adjust to feedback when they presented a learning curve, moving gradually away from risky choices toward safer ones in the course of the trials. In 2 of 4 studies using the IGT MS patients showed a lower learning-index (50%). In another 5 IGT-based measurements, where the index was not explicitly calculated, MS patients had a learning curve that was less steep than HCs or showed a more pronounced impairment in the last blocks of the IGT, indicating slowed learning from feedback.

Moreover, MS patients had longer deliberation times in two of 3 altogether studies.

In total, in 11/17 (64.7%) of cases, qualitative changes in DM were seen in MS patients compared to HCs.

#### 3.1.2. Association between Decision-Making and Cognitive Dysfunction or Physical Disability

Nine studies including 12 separate tasks of DM assessed correlations of performance in other cognitive domains with results in the DM tasks (Tables 1 and 2). A total of 6/12 (50%) of tasks showed a correlation with any kind of cognitive performance (Table 3). More precisely, 4/12 (33.3%), correlated with a measurement of executive functioning, whereas processing speed and attention capacity were correlated with DM performance in 5/9 times (56%). DM under ambiguity correlated in 2/11 (18%) cases with measurements of other cognitive functions, DM under risk in 9/16 (56%) (Table 4). *χ*^2^ showed a significant difference between the two types of DM (*p* = 0.04792), suggesting that DM under risk is more sensitive to cognitive impairment than DM under ambiguity.

Concerning physical disability, patient groups with a median/mean EDSS lower than 3 performed worse than HC in 54.5% (6/11) of decision-making tasks. In patients with EDSS ≥ 3, 80% (4/5) of DM scores were worse in MS patients than HC (*χ*^2^ between both groups being nonsignificant; *p* < 0.33).

In tasks measuring DM under risk, all tasks in patients with an EDSS > 2 showed an impairment. On the other hand, DM under ambiguity (IGT) was preserved even in a patients group with a mean EDSS of 7.

#### 3.1.3. Association between Decision-Making and Emotional Reaction

Emotional reactivity was measured using skin conduction reactivity (SCR) in only two studies. SCR was reduced in one study reporting DM under ambiguity [20] but preserved in the other, where DM was taken under risk [26]. Reduced expression of negative emotion, such as disappointment and regret, was stated in MS patients when confronted with negative outcomes [26].

## 4. Discussion

The aim of our study was to evaluate the decision-making (DM) abilities of patients with MS. Given the complexity of the process, we further considered the relationship between DM and overall cognitive performance as well as emotional components to the DM process. On the basis of 12 eligible studies, we found decreased DM performance in the MS group in 64.7%. Our results indicate an overall alteration of DM abilities in MS patients, since both DM under ambiguity and DM under risk were affected. DM under risk was altered in 67% and DM under ambiguity in 64% of measurements in MS patients with no statistical difference in the performance between the two task groups.

Further analysis revealed also differences between MS patients and HCs in qualitative measurements such as reaction times or reaction to feedback through most tasks (11/17), even when the groups reached comparable net results. Only one study [24] showed a preserved learning curve in the IGT. Notably, the subjects in this study had a low EDSS and short disease duration. These findings suggest a gradual decline of the learning process over the course of the disease. Hence a qualitative evaluation of the learning process might reflect a more sensitive rating of DM performance than the net scores do.

DM performance in general correlated with ventricular size as well as white matter lesions [21, 23] and is influenced by lesions in the temporal region [28].

Functional studies found decision-making-related activity in the insular, prefrontal, and frontal lobes as well as the cingulate gyrus and caudate nuclei [21, 32]. More specifically, the stage of deliberation and choice-making correlated with activity of a large network, involving the medial and dorsolateral prefrontal lobe, middle frontal gyrus, anterior cingulate, caudate [21], and insula [32].

Feedback processing and risk adjustment were associated with the hippocampus [21] and orbitofrontal areas [32].

With our results confirming an alteration of DM performance in a majority of MS patients, the question on the underlying mechanism arises. In IGT, where the index of risky choices is obtained by subtracting the number of disadvantageous or “risky” choices from the advantageous choices, a lower index indicates risk-seeking behaviour. The results here consistently pointed to MS patients showing a risk-seeking tendency. This could be due to hypersensitivity to reward or to a reduced ability to weigh immediate gain against long-term outcome, a so-called “myopia for the future.” The study by Nagy and colleagues [22] addressed this question specifically. They used two different versions of the IGT: the standard ABCD version and an adapted EFGH version, where subjects receive punishment with each card but varying reward. Impaired performance in the standard IGT indicates increased sensitivity to reward, whereas in the EFGH version it points to behaviour of risk avoidance. MS patients showed impaired decision-making in both versions of the task, suggesting a general deficit to evaluate long-term outcomes in DM under ambiguity.

However, MS patients have demonstrated risk-aversive behaviour in tasks using DM under risks paradigms, such as the WOF [26] and longer deliberation time in the CGT [21, 26]. Such results, specifically found in these tasks, have been interpreted as both adaptive changes in the face of cognitive deficits and/or alterations in emotional reactivity due to MS, when a decision has to be taken under risk.

We could not demonstrate an impact of severity of MS, as measured by the EDSS on DM performance, possibly because of an insufficient size. However, qualitative analysis, comparing groups with an EDSS of under and over 3, suggests an influence of disease progression. On the other hand, one group with an EDSS of 7 [16] showed a preserved IGT performance. Notably, this group consisted of patients with a secondary progressive course of MS, raising the question of the influence of the disease course on DM abilities. Only three studies assessed DM performance between different MS courses and came to contradictory conclusions. However, when looking at measurements of patients with a relapsing remitting MS only (*n* = 12 in 8 different studies), the proportions of failures were comparable to the MS group as a whole (8/12 impaired, 66.6%).

A correlation of a high relapse rate with DM performance has been described [24]. This could be an explanation for the inconsistent performance pattern, especially regarding the correlation with EDSS and IGT. Patient groups with low EDSS scores might also include patients with more aggressive form of MS, presenting a higher relapse rate and therefore potentially associated with DM alterations despite low functional impairment. Also, in one study, higher EDSS correlated with higher anxiety and less impaired IGT score [20]. Possibly, the anxiety in more affected patients led to risk-aversive behaviour, thus attenuating risk-taking behaviour on performance in the IGT.

As stated in the introduction, cognitive and emotional functionalities are important for a normal performance in DM tasks. These aspects will now be discussed separately.

### 4.1. Association with Cognition

DM tasks require attentional, mnestic, executive, and prospective thinking and learning abilities. Consequently, in 50% (6/12) of the evaluated studies, the results correlated with the performance in other cognitive domains, mostly with processing speed and attention capacity. Hence, the evaluated studies corroborate previous findings, where cognitive dysfunctions had a negative, though not exclusive impact on DM.

Of the DM tasks, the IGT was least correlated with other cognitive measures (see Table 4). This is in line with previous results, showing DM under ambiguity less dependent on general cognitive performance than DM under risk [3].

### 4.2. Emotion

The emotional component of the DM process was assessed in only a few of the studies and a quantitative analysis was therefore not possible. We will, however, do a qualitative evaluation.

In the IGT, healthy subjects generate anticipatory SCR before choosing cards from a “risky” deck even before consciously understanding the rules of the game, thus reflecting their somatic state activation. This part of the IGT has been called the “hunch period” [33]. Kleeberg and colleagues [20] described reduced SCR and a later switch to good decks in MS patients with an EDSS > 2, possibly skipping “hunch” period and switching to good decks only after overtly understanding the rules. This would indicate a disruption of the emotional component of a DM process. Another study reported reduced expression of negative emotions after counterfactual information but normal SCRs in DM under risk [26]. Possible explanations for the divergence between the SCR results in the two studies are the differences in EDSS and disease duration. As discussed for general DM performance, emotional components of DM might also decline during the disease course. Alternatively, the difference could be due to DM under ambiguity being more closely associated with the emotional aspects of DM, while DM under risk seems more related to other cognitive functions. Further research should be conducted to investigate this possible dissociation between the two forms of DM.

Fatigue was not mentioned as a possible correlated symptom in most of the studies. The only two studies assessing it stated elevated fatigue levels in MS patients compared to HCs [23, 24]. However, no study correlated fatigue with DM performance. Fatigue is a common symptom in MS and likely to influence the performance on cognitive tests, especially when sustained mental effort is required [13]. It was only rarely assessed in the studies and neither was always stated if the DM task were administered before or after the often extensive neuropsychological testing. Therefore, an influence of fatigue on the results of the DM tasks cannot be excluded.

There are a certain number of limitations to this paper. Group sizes as well as demographic constellation varied across the included studies. Also, there were methodological differences in the studies regarding the tests administered and the outcomes measured. However, the aim of this systematic review being a descriptive overview on existing literature has in our eyes been achieved.

## 5. Conclusions

Taken together, our review of the existing studies suggests the presence of qualitative as well as quantitative changes in decision-making abilities in the majority (2/3rd) of MS patients by showing altered decision-making performance in tasks under risk and under ambiguity. Decision-making performance, especially under risk, might be influenced by disease progression, but performance by MS patients was independent of other cognitive measures in half of the analysed decision-making tasks. Thus, decision-making is an aspect of cognition to be kept in mind by doctors and nurses treating MS patients, even in the absence of other cognitive deficits.

## Figures and Tables

**Table 1:** Tests used to assess cognitive domains other than decision-making.

Executive functioning	IQ	Processing speed and attention capacity	Memory
Shifting: (i) Trail Making Test B (TMT-B)	National Adult reading test (NART, premorbid IQ)	Symbol Digit Modalities Test (SDMT)	Rey auditory verbal learning test (RAVLT)

Inhibition (i) Hayling sentence completion (ii) Stroop time	Wechsler Adult Intelligence Scale-III (WAIS-II, current IQ)	Trail Making Test A (TMT-A)	Selective reminding test (SRT)

Updating: (i) Verbal fluency (letter and category) (ii) Digit span backwards		9-hole Pegboard	Adult Memory and Information Processing Battery (AMIPB story/figure)

Planning: (i) Wisconsin Card sorting test (ii) Modified card sorting test (MCST) (iii) Stockings of Cambridge (SOC) (iv) Tower of Hanoi (v) Tower of London		Digit span forward	10/36 spatial recalling

Behavioural Assessment of the Dysexecutive Syndrome (BADS)		Paced Auditory Serial Addition (PASAT)	7/24 spatial recalling
(i) Raven's coloured progressive matrices			

**Table 2:** Studies comparing decision-making in MS patients and HCs and baseline data of their respective cohorts.

Cohort	*n* MS/CIS	*n* HC	EDSS	MS-type	Disease duration (mean (SD))	Age in years (mean (SD))	Affective changes	Cognitive changes	Behavioural changes	Fatigue in MS patients
Farez et al. 2014 [18]	27	27	Mean: 1.03 (SD 0.8)	RR	7.9 months (8.1)	33.3 (9.7)	−	+	Nm	Nm

Simioni et al. 2012 [26]	72	38	Mean: 1.9 (SD: 0.5, range: 1.5–3)	RR	5.06 years (3.3)	34.6 (6.3)	+	+	−	Nm

Muhlert et al. 2014 [21]	105	43	Median: 5 (range: 0–8.5)	RR (*n* = 61), SP (*n* = 26), PP (*n* = 18)	14.9 years (9.6)	45.9 (10.4)	+	+	Nm	Nm

Radomski et al. 2015 [23]	32	20	Median: 3 (range: 0–6.5)	RR (*n* = 22), SP (*n* = 10)	15.9 years (10.3)	50.8 (9.5)	−	+	+	+

Kleeberg et al. 2004 [20]	20	16	Median: 2 (range: 1.5–6.5)	RR (*n* = 16), SP (*n* = 4)	Median 103 months (range: 7–300)	Median 38 (25–57)	+	+	+	Nm

Cogo et al. 2014 [17]	60	35	Mean 1.4 (SD: 1, range: 0–3.5)	RR	40.8 months (SD 45, range: 2–264)	34 (7.7)			nm	Nm

Simioni 2008 et al. [24]	109/56	50	MS: Mean 1.82 (SD: 0.4)	RR (*n* = 109),	2.8 (1.9) years	34 (9.3)	+	+	+	+
			CIS: Mean: 1.66 (SD: 0.4)	CIS (*n* = 56)	1.4 (1.3) years	37.9 (8.3)	+	+	+	+

García-Molina et al. 2008 [19]	18	18	Mean: 6.7 (SD: 0.69)	RR (*n* = 7); SP (*n* = 4); PP (*n* = 7)	17.14 (6.55) years	49.33 (7.8)	Nm	Nm	Nm	Nm

Nagy et al. 2006 [22]	21	30	Mean: 1.7 (range: 0–3)	RR	3.1 years (1.1)	32.4 (9.8)	Nm	+	Nm	Nm

Roca et al. 2008 [27]	12	12	<2	RR	Mean 29.5 months (SD 12.4, range: 11–36)	32.5 (7.97)	+	+	Nm	Nm

García-Molina et al. 2009 [16]	9	18	Mean: 7.2 (SD: 0.3)	PP	17.3 years (6.1)	51.4 (4.7)	Nm	Nm	Nm	Nm
	10	18	Mean: 7 (SD: 0.5)	SP	16.6 years (4.8)	46 (14.1)	Nm	Nm	Nm	Nm

Azcárraga-Guirola et al. 2016 [28]	16	19	Range: 2.5–4.5	RR (*n* = 9); SP (*n* = 4); not defined (*n* = 3)	8.9 years (7)	39.4 (12.6)	−	+	Nm	Nm

Nm: not measured; +: present; −: absent; HC: healthy controls; RR: relapsing remitting; SP: secondary progressive; PP: primary progressive; SD: standard deviation.

**Table 3:** Decision-making performance in MS patients compared to HCs and correlations with other cognitive domains.

Study	Measurement	Altered DM net scores (*z*-score)	Qualitative changes in DM	Correlation of DM with any measure of cognition
Farez et al. (2014)	GDT	+ (2.76)	−	+
	IGT net	+ (4.39)	+	nm
Simioni et al. (2012)	WOF	+	+	−
	CGT	− (3.75)	+	+
Muhert et al. (2014)	CGT	+	+	+
Radomski et al. (2015)	GDT	+ (0.75)	−	+
Kleeberg et al. (2004)	IGT net	− (0.3)	+	+
Cogo et al. (2014)	GDT	− (0.22)	−	−
	IGT net	− (0.31)	−	+
Simioni et al. (2008)	IGT net	− (0.1)	−	−
García-Molina et al. (2008)	IGT	+ (1.02)	+	nm
Nagy et al. (2006)	IGT (ABCD)	+	+	−
	IGT (EFGH)	+	+	−
Roca et al. (2008)	IGT	+ (0.75)	+	nm
García-Molina et al. (2009)	IGT (SP)	− (0.38)	+	nm
	IGT (PP)	+ (1.02)	+	nm
Azcárraga-Guirola et al. (2016)	IGT	+ (0.7)	−	−

Total		11/17	11/17	6/12

+: present, −: absent, nm: not measured, SP: secondary progressive, PP: primary progressive, numbers in brackets: *z*-values.

**Table 4:** Proportion of studies showing a correlation between DM tasks and other cognitive domains.

	Executive function	IQ	Processing speed and attention capacity	Memory	Total
IGT	1/6	-	1/4	0/1	2/11
GDT	1/3	-	2/3	1/3	9/16
WOF	0/1	-	-	-	
CGT	2/2	0/1	2/2	1/2	

Total	4/12 (33%)	0/1 (0%)	5/9 (56%)	2/6 (33%)	
